# Mechanisms and clinical application potential of mesenchymal stem cells-derived extracellular vesicles in periodontal regeneration

**DOI:** 10.1186/s13287-023-03242-6

**Published:** 2023-02-13

**Authors:** Liangrui Chen, Shasha Zhu, Shujuan Guo, Weidong Tian

**Affiliations:** 1 State Key Laboratory of Oral Disease & National Clinical Research Center for Oral Diseases & National Engineering Laboratory for Oral Regenerative Medicine, West China School of Stomatology, Sichuan University, Chengdu, 610041 People’s Republic of China; 2 Department of Oral and Maxillofacial Surgery, West China Hospital of Stomatology, Sichuan University, Chengdu, 610041 Sichuan People’s Republic of China; 3 Department of Periodontics, West China School of Stomatology, Sichuan University, Chengdu, 610041 Sichuan People’s Republic of China

**Keywords:** Periodontitis, Extracellular vesicles, Mesenchymal stem cells, Periodontal regeneration

## Abstract

Periodontitis is a high prevalence oral disease which damages both the hard and soft tissue of the periodontium, resulting in tooth mobility and even loss. Existing clinical treatment methods cannot fully achieve periodontal tissue regeneration; thus, due to the unique characteristics of mesenchymal stem cells (MSCs), they have become the focus of attention and may be the most promising new therapy for periodontitis. Accumulating evidence supports the view that the role of MSCs in regenerative medicine is mainly achieved by the paracrine pathway rather than direct proliferation and differentiation at the injured site. Various cells release lipid-enclosed particles known as extracellular vesicles (EVs), which are rich in bioactive substances. In periodontitis, EVs play a pivotal role in regulating the biological functions of both periodontal tissue cells and immune cells, as well as the local microenvironment, thereby promoting periodontal injury repair and tissue regeneration. As a cell-free therapy, MSCs-derived extracellular vesicles (MSC-EVs) have some preponderance on stability, immune rejection, ethical supervision, and other problems; therefore, they may have a broad clinical application prospect. Herein, we gave a brief introduction to MSC-EVs and focused on their mechanisms and clinical application in periodontal regeneration.

## Introduction

Periodontitis is a dental plaque-caused chronic inflammatory disease invading periodontal tissue. It is distinguished by pathologic loss of alveolar bone and the periodontal ligament (PDL), thus causing tooth mobility and even adult tooth loss [1]. Although the present clinical treatment, including scaling and root planning, periodontal flap surgery, and guided tissue regeneration (GTR), can control and restore the periodontal tissue defect to a certain extent, it still cannot achieve ideal results for periodontal regeneration, in which cementum, PDL, and alveolar bone reconstruction are required [2]. The development of tissue engineering and regenerative medicine has a history of more than three decades. Periodontitis-caused tissue destruction is unique given the specificity of the local inflammatory microenvironment and oral environment. The reformation of oriented PDL fibers and their firm attachment to the newly formed cementum and alveolar bone is still significant challenges. Novel approaches need to be developed to achieve ideal therapeutic outcomes for patients with periodontitis.

MSCs are multipotent stem cells with the ability to self-renew and differentiate in multiple directions [3]. The tissue regeneration process is associated with cell migration, proliferation, differentiation, extracellular matrix remodeling, as well as angiogenesis and osteogenesis [4–7]. Due to their unique differentiation potential, hematopoietic support and immune regulation, MSCs have been widely used as a cell therapy for a variety of diseases [8]. In periodontal tissue, periodontal ligament stem cells (PDLSCs) and dental follicle stem cells (DFSCs) are the leading candidate MSCs for periodontal regeneration, for their strong ability to form cementum, PDL-like structures and alveolar bone [9–11]. By performing local transplants, other non-periodontal-derived MSCs such as dental pulp stem cells (DPSCs), bone marrow stem cells (BMSCs), and adipose tissue-derived stem cells (ADSCs) also aid in the regeneration of periodontal tissue [12].

However, when employing MSC transplantation to treat tissue defects, the research found that: less than one percent of MSCs could home and colonize in the tissue defect area, continuously promoting tissue regeneration, and the paracrine mechanism of MSCs plays a more important role than the replacement of damaged cells by differentiation [13]. In this process, MSCs can release varieties of bioactive molecules (such as growth factors, cytokines, chemokines, etc.) through the paracrine mechanism, which has a crucial impact on the control of biological processes. EVs, a group of nano-sized particles, are important paracrine products that engage in communication between cells [14]. The surface molecules of EVs enable them to target recipient cells. After attaching to target cells, EVs can change the physiological state of recipient cells through various pathways. They may mediate signal transduction through receptor-ligand interactions, internalization through endocytosis, or fusion with the cell membrane of the target cell to deliver their contents into the cytoplasm of the recipient cell [15]. In these ways, they can regulate cell proliferation, differentiation and apoptosis [14]. Besides, they can also modulate immune function, promote angiogenesis and osteogenesis, and thus induce tissue regeneration [7, 16]. At present, EVs have been widely used in regenerative medicine as cell-free therapy.

As for MSCs, though they are excellent seed cells utilized for tissue regeneration, some non-ignorable problems exist in their clinical application. They have a low survival rate after in vivo transplantation and may have unpredictable cell growth and differentiation after transplantation [17]. Autologous MSCs have limited sources, while allogenic MSCs may have tumorigenicity or induce host immune reactions. Conversely, the application of EVs can subtly avoid these problems and outperforms stem cell therapy in terms of safety and versatility [18]. EVs are low immunogenic, cost-effective, stable, and can be stored for a considerable amount of time without hazardous chemicals like dimethyl sulfoxide (DMSO). Besides, EVs are similar to traditional pharmaceutical preparations and involve fewer ethical issues than stem cells, so evaluating safety and effectiveness is less complicated. In addition to being used for disease treatment by themselves, EVs can be modified and redesigned as natural gene and drug carriers, and even their cargos can be customized as needed [19]. According to the above, MSC-EVs do have some advantages over MSCs in clinical application, making it promising for clinical translation.

In this review, MSC-EVs are briefly introduced, including their sources, biogenesis, and molecular composition. The current progress of studies on their mechanisms in promoting periodontal regeneration and their application forms are highlighted. At last, based on existing clinical research and relevant patents, we discussed the potential of bench-to-bed translation and existing challenges to provide a basis for further clinical application.

## Extracellular vesicles

EVs produced by cell secretion are spherical membranous vesicles formed by a lipid bilayer, which are highly heterogeneous. They are classified based on their molecular diameters, which range from 30 to 5000 nm [20]. Initially, these particles were thought to remove metabolic waste from cells. Not until the late 1990s did the biological function of EVs gain attention from researchers [21]. EVs secreted by MSCs act as intercellular communication mediators to regulate cellular biological activities of recipient cells, which is a pivotal step in regenerative medicine.

### Biogenesis

"EVs" is a general term for a large group of substances. It is worth noting that the size ranges overlap among different subtypes of EVs, so the mechanism of biogenesis remains the chief difference in distinguishing them. Here, we give a brief overview of the biogenesis of the three main subtypes.

The biogenesis of exosomes (30–150 nm) requires endosomal systems [22]. First, early endosomes form and fuse with endocytic vesicles at the start of endocytosis. Next, multivesicular bodies (MVBs) are produced through either endosomal sorting complexes required for transport (ESCRTs)-dependent or ESCRTs-independent mechanisms [23–25]. Intraluminal vesicles (ILVs) accumulated by MVBs inward budding, which is also known as late endosomes, are taken for the beginning of exosome biogenesis. Microvesicles (MVs) (100–1000 nm) are released directly by cell membrane budding and fission. Many factors lead to this process, such as phospholipid repartitioning and cytoskeletal protein contraction [26]. Unlike exosomes and MVs, apoptotic bodies (ApoBDs) (1000 nm-5 μm) are discharged when a cell undergoes apoptosis [27]. Besides mediating intercellular communication, ApoBDs also contribute to apoptotic cell clearance [28].

To summarize, MSC-EVs can be generated by diverse mechanisms and then released from various cells, performing a variety of biological functions such as intercellular messenger.

### Molecular composition

The heterogeneity of EVs and different purification methods make the result of composition analysis controversial [29], and no clear consensus has been reached yet [30]. Generally, EVs consist mainly of proteins, lipids, DNA and RNA [22, 31].

Proteins participate in EVs biogenesis and promote the interaction of EVs and receptor cells [32]. Proteins in MSC-EVs are also essential in immune regulation [33] and inflammatory reaction [34] and may be partially involved in disease regulation [35, 36]. Lipids are fundamental components of the EV membranes. The differences in lipid composition among EV subtypes reflect their different biogenesis. As for nucleic acids, DNA composition in EVs is involved in intercellular communication [37], cellular homeostasis [38], and genomic evolution [39, 40]. Except for serving as biomarkers for disease diagnosis [41], EV-RNAs can participate in a variety of cellular pathways by targeting transcription factors and genes, according to RNA-seq analysis by Eirin et al. [42].

The contents of EVs are distinct due to the formation of vesicles, the types of cells from which the vesicles are derived, and the environmental conditions. In addition, specific drugs can be carried in some engineered EVs for therapeutic applications [43]. Pretreatment of MSCs can affect the contents and function of their EVs. For example, the paracrine, regenerative, and repair capabilities of MSCs significantly enhanced after LPS pretreatment, which has emerged as a potential therapy for inflammatory diseases and tissue injury [44]. In periodontal regeneration, scholars have shown that EVs secreted from DFSCs after 250 ng/ml LPS preconditioning manifested better reduction in *Porphyromonas gingivalis* pathogenicity and inhibition of bone loss compared with the controls [45–47]. This may be related to the altered content of bioactive molecules in EVs. Zheng et al. pretreated PDLSCs with 1 μg/ml LPS and found decreased expression of miR-155-5p and upregulated expression of Sirtuin 1 in their secreted exosomes, alleviating the inflammatory microenvironment [48]. In addition to LPS, TNF-α pretreatment of MSCs has similar effects. In the study by Nakao et al., TNF-α pretreatment caused GMSCs to secrete exosomes containing more miR-1260b, which were able to interfere with the expression of Wnt5a mRNA in receptor cells, thereby inhibiting bone resorption [49]. Moreover, gene modification of MSCs directly regulates the expression of paracrine products. According to Xu et al., exosomes derived from P2X7R gene-modified PDLSCs showed high expression of miR-3679-5p, miR-6747-5p, and miR-6515-5p, and they rescued the dysfunction of inflammation-impaired PDLSCs by binding to GREM 1 protein [50].

Therefore, the composition of EVs not only reflects the characteristics of their origins but also is inseparable from their function. Taking advantage of this feature, artificially altering EVs contents to exert tissue regeneration may be a promising research direction.

## Mechanisms of MSC-EVs promoting periodontal regeneration

**Fig. 1:**
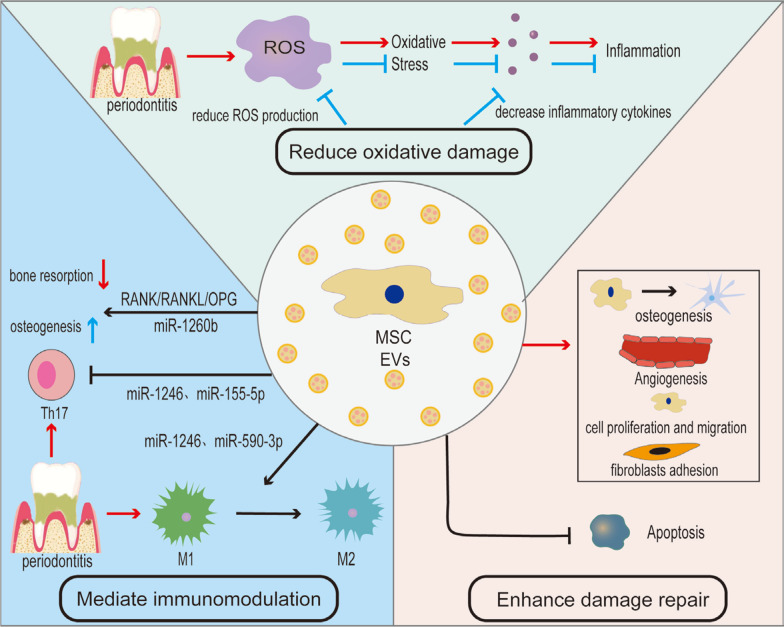
Schematics of mechanisms that MSC-EVs promote periodontal regeneration

MSCs release vectors such as EVs through a paracrine mechanism to mediate intercellular communication, activate target cell signaling pathways, and affect the biological activities of cells in a specific microenvironment, thereby inducing regeneration of damaged tissues (Fig. 1). Although it remains to be fully understood how MSC-EVs promote periodontal regeneration, lots of studies have been conducted recently on the underlying mechanisms (Table 1), which will be specifically explained below.

**Table 1:** Studies of mechanisms that MSC-EVs promote periodontal regeneration

Cell source	Cell type	Study model	Active molecules of EVs	Mechanisms	Effects	References
Non-odontogenic stem cells	BMSCs	In vitro	-	Reduce oxidative damage	Alleviate the pro-inflammatory state of macrophages induced by Pg-LPS	Ye et al. [52]
		In vivo (rat model) & in vitro	-	Mediate immunomodulation	Facilitate periodontal tissue regeneration	Liu et al. [59]
	MSCs	In vivo (rat model) & in vitro	-	Enhance damage repair	Enhance periodontal regeneration	Chew et al. [70]
	ADSCs	In vivo (rat model)	-		Have immunomodulatory and anti-inflammatory effects	Mohammed et al. [72]
Odontogenic stem cells	PDLSCs	In vitro	-	Reduce oxidative damage	Display anti-inflammatory and immunosuppressive effects	Čebatariūnienė et al. [57]
		In vivo (murine model) & in vitro	miR-590-3p	Mediate immunomodulation	Alleviated periodontal inflammatory injury and macrophage pyroptosis	Han et al. [61]
		In vitro	miR-155-5p		Alleviate inflammatory microenvironment	Zheng et al. [48]
		In vitro	miR-6515-5p	Enhance damage repair	Change the local inflammatory microenvironment and improve tissue regeneration	Xu et al. [50]
		In vivo (rat model) & in vitro	-		Facilitate pro-angiogenesis	Zhang et al. [69]
	GMSCs	In vivo (rat model) & in vitro	-	Reduce oxidative damage & mediate immunomodulation	Facilitate periodontal tissue regeneration	Zarubova et al. [53]
		In vitro	-		Reduce the inflammatory response	Zhang et al. [54], Wang et al. [55]
		In vivo (mouse model) & in vitro	exosomal miR-1260b	Mediate immunomodulation	Inhibit periodontal bone loss	Nakao et al. [49]
	DPSCs	In vivo (mouse model) & in vitro	miR-1246	Reduce oxidative damage & mediate immunomodulation	Promote alveolar bone and periodontal epithelium healing	Shen et al. [58]
		In vivo (mouse model) & in vitro	miR-1246	Mediate immunomodulation	Enhance anti-inflammatory property	Zhang et al. [64]
		In vitro	miR-378a	Enhance damage repair	Enhance cell angiogenesis	Zhou et al. [67]
	DFSCs	In vivo (beagle dog model) & in vitro	GSR and SOD1	Reduce oxidative damage & mediate immunomodulation	Inhibit alveolar bone loss and enhance periodontal regeneration	Huang et al. [46]
		In vivo (rat model) & in vitro	-	Enhance damage repair	Contribute to alveolar bone regeneration	Yi et al. [66]
		In vivo (rat model) & in vitro	-		Promote periodontal tissue regeneration	Ma et al. [71]
		In vivo (rat model) & in vitro	-		Promote periodontal regeneration in an inflammatory microenvironment	Shi et al. [47]
	SHED	In vivo (mouse model)	-	Reduce oxidative damage & enhance damage repair	Enhance the osteogenic ability of BMSCs	Wei et al. [56]
		In vitro	Wnt3a and BMP2	Enhance damage repair	Enhance the osteogenic ability of PDLSCs	Wang et al. [65]

### Play an active role in reducing oxidative stress damage

In recent years, periodontal tissue damage caused by oxidative stress created by excess reactive oxygen species (ROS) has received extensive attention [51]. ROS can damage periodontal tissue directly or indirectly during periodontitis. The direct effect refers to the cytotoxicity of ROS, that is, the oxidation of lipids, proteins, and DNA in cells and extracellular matrix, or the stimulation of extracellular matrix degradation by matrix proteases. Indirect effects include causing an inflammatory response and impairing the immune system.

MSC-EVs alleviate oxidative stress injury mainly by enhancing resistance to ROS. Based on the protein spectrum sequencing of DFSC-sEV with and without LPS treatment, Huang et al. found that EVs themselves carry antioxidants, directly neutralize ROS, and inhibit the ROS/JNK pathway, thus playing an antioxidant mechanism [46]. Increased oxidative stress may elicit the release of inflammatory cytokines from immune cells, exacerbating the inflammatory response. EVs can also decrease inflammatory cytokines (IL-6, IL-1β, IFN-γ, TNF-α, etc.) secreted by both immune cells and periodontal tissue cells [52–56], which further aggravates the inflammatory response and exacerbates alveolar bone resorption through osteoimmune.

Studies conducted both in vivo and in vitro demonstrated that EVs inhibited nuclear factor kappa B (NF-κB) activation, which are key regulators of inflammation and oxidative stress, thereby downregulating the inflammatory response. In the study of ČEBATARIŪNIENĖ et al., EVs permanently inhibited NF-κB activity in basal and LPS-induced PDLSCs by increasing the phosphorylation of Akt and its downstream target protein glycogen synthase kinase-3β (GSK-3β), without affecting PDLSCs osteogenic mineralization [57]. What’s more, SHEN et al. found that the inflammatory response is down-regulated by NF-κB and P38 MAPK signaling pathways inhibition induced by DPSC-exosomes [58].

### Mediate immunomodulation of periodontal tissue

The immunomodulatory function of MSC-EVs is a key characteristic of their clinical application. EVs from various origins have varying impacts on the course of infectious diseases. In periodontitis, MSCs-EVs participate in immune regulation through different mechanisms and can promote the immune microenvironment from one that is pro-inflammatory to one that is anti-inflammatory, which is conducive to tissue repair and regeneration.

Elucidating the connection between bone cells and the immune system, which is referred to as osteoimmunology, helps to understand the pathogenesis of periodontitis. The RANK/RANKL/OPG pathway is one of the cardinal signaling pathways of periodontal osteoimmune. The receptor activator of NF-κB ligand (RANKL) is the primary regulator of osteoclast formation, the mRNA and protein levels of which are related to the severity of periodontitis in patients. RANK is the receptor of it. Osteoprotegerin (OPG), as a decoy receptor, prevents the bone from binding to RANK by binding to RANKL. Studies have confirmed that MSC-EVs can participate in the RANK/RANKL/OPG pathway in an inflammatory environment and inhibit excessive bone resorption caused by osteoclast activation [46, 59]. In NAKAO et al.’s research, miR-1260b, which targets the Wnt5a-mediated RANKL pathway, was up-regulated by gingival mesenchymal stem cells (GMSC)-derived exosomes pretreated with TNF-α, thereby inhibiting the osteoclastic activity [49].

Regulation of osteoimmune is inseparable from immune cell networks. Among them, macrophages along with other immune cells are pivotal in the progression of periodontitis. Macrophage (Mø) polarization is a reversible process influenced by many factors, and two different phenotypes were described: one is the pro-inflammatory phenotype (M1), and the other is the anti-inflammatory phenotype (M2), also known as the wound-healing phenotype. The phenotypic conversion of macrophages from M1 to M2 is proved to promote the periodontal tissue regeneration and inhibit alveolar bone loss [60]. MSC-EVs can promote macrophage polarization from M1 to M2 through different signaling pathways under the inflammatory microenvironment [46, 55, 59]. Zhang et al.'s in vitro experiment proved that GMSC-exosomes have the same polarization-promoting effect even under a high-lipid microenvironment while regulating lipid balance [54]. Shen et al. found that DPSC-exosome/CS promoted Mø polarization toward M2-type in periodontitis mice, the mechanism of which may be related to miR-1246 in DPSC-exosomes [58]. Besides, the process of macrophage death by pyroptosis triggers inflammation. According to Han et al., miR-590-3p carried by hPDLSC-EVs inhibited the transcription of TLR4 in macrophages, and then, their pyroptosis was reduced, thus alleviating inflammatory damage [61].

In addition to macrophages, the immunoregulatory role of T cells in periodontitis cannot be underestimated. T-helper 17 (Th17) cells and regulatory T (Treg) cells are common subsets of CD4 + T cells. Th17 cells are the only T cells that can damage bones and drive osteoclast development, which is essential for host defense. Treg cells prevent too aggressive or protracted immune responses, which successfully inhibit osteoclastogenesis [62]. Periodontitis progression in animal models (mice and canines) is suppressed when Treg accumulation is induced in periodontal tissue [63]. MSC-EVs are proven to restore inflamed periodontal tissue both in vivo and in vitro [53]. MiR-1246 in DPSC-3D-exosomes [64] and miR-155-5p in PDLSC-exosomes [48] are key active molecules found by researchers that contribute to this process. The former inhibits Nfat5 expression and mediates the polarization of reactive Th17 cells in a sequence-dependent manner, while the latter down-regulated Th17 cells through miR-155-5p/SIRT1 pathway. Both of them showed enhanced anti-inflammatory effects.

### Enhance periodontal tissue damage repair

The ultimate objective of periodontitis therapy is to accomplish periodontal tissue repair and regeneration, the key to which is the formation of functional soft and hard tissue composite structures, namely alveolar bone regeneration and orientation of new PDLs. At the cellular level, osteogenesis, angiogenesis, periodontal cell migration, proliferation, and differentiation into several different sorts of cells are required, among which osteoblasts and fibroblasts are of great importance.

MSC-EVs carry bioactive molecules and interact with cells around the tissue defect area, which can induce their osteogenic differentiation. Exosomes derived from stem cells from exfoliated deciduous teeth (SHED) are proved to enhance the osteogenic ability of periodontal tissue cells. In addition to enhancing the osteogenic ability, SHED-exosomes can also inhibit the apoptosis of BMSCs and reduce their adipogenicity in the in vivo experiments of Wei et al. [56]. Furthermore, Wang et al. verified in vitro that Wnt3a and BMP2 in SHED-exosomes activated the BMP/Smad and Wnt/β-catenin signaling pathways; then, PDLSC osteogenic properties were improved by enhancing smad1/5/8 phosphorylation and nuclear β-catenin protein expression [65]. Xu et al. found through miRNA sequencing that miR-6515–5 was differentially expressed in exosomes secreted by PDLSC after P2X7R modification. miR-6515-5 inhibited the expression of target gene GREM 1 through the TGF/BMP signaling pathway, thereby significantly promoting osteogenic differentiation [50]. Likewise, in vitro studies by Yi et al. showed that EVs could also promote the osteogenic capacity of DFSCs by activating the PLC/PKC/MAPK pathway [66].

The periodontal tissue regeneration process is complex for the reason that periodontal wounds heal on nonvascular and nonvital hard tissues. The regeneration of alveolar bone is not only related to the binding of a series of osteogenesis-related molecules (such as BMP9, TGF 1, RUNX2, osteocalcin, etc.) to calcium, phosphorus, and collagen molecules in the extracellular matrix but also requires a large amount of angiogenesis to ensure adequate blood supply. When periodontitis causes progressive inflammation, abnormal angiogenesis tends to occur, and EVs can improve the vascular function of newly formed PDL under such conditions. Zhou et al. created a periodontitis-impaired dental pulp stem cell model in vitro, and EVs derived from it carried miR-378a, which promoted endothelial cell angiogenesis by activating the Hedgehog/Gli1 signaling pathway and downregulating Sufu [67]. Vascular endothelial growth factor (VEGF) is considered a major controller of angiogenesis, and it promotes the survival, proliferation, and migration of vascular endothelial cells [68]. In the study of Zhang et al., exosomes secreted by PDLSCs enhanced human umbilical vein endothelial cells (HUVEC) angiogenesis by transferring Vascular Endothelial Growth Factor A (VEGFA), which served as the crucial communicator between these two types of cells [69]. This is because the microenvironment of periodontal inflammation inhibited the expression of miR-17-5p in PDLSCs and reduced its targeting of VEGFA, resulting in a high expression of VEGFA. Exosomes transported highly expressed VEGFA from PDLSCs to HUVECs, promoting their proliferation, and as a result, angiogenesis of periodontal ligaments was enhanced.

The biological function of periodontal tissue cells is crucial in periodontal regeneration and repair. DFSC solution digested by collagenase was acquired by Yi et al. to get collagenase-released MVs (CRMVs) [66]. DFSC proliferation, migration, and osteogenic differentiation were successfully enhanced by CRMVs in vitro, and PLC/PKC/MAPK pathways may be involved. EVs can enhance PDLSC proliferation, migration, and osteogenic differentiation in an inflammatory microenvironment. This may be related to some signaling pathways involved in EVs, such as the pro-survival AKT and ERK signaling pathways activated by C73-mediated adenosine receptors [70] and the activated p38 MAPK signaling pathway [71]. Shi et al. believed that this was also related to the reduction in the RANKL/OPG ratio by EVs to a certain extent [47]. Furthermore, EVs also support the adhesion and migration of fibroblasts and form new PDL attachments by recruiting periodontal ligament fibroblasts. For instance, ADSC-exosomes can promote the migration, proliferation, and other biological functions of fibroblasts as well as endothelial angiogenesis in the rat model [72].

It is tough to elucidate the regeneration effect mediated by EVs. The biological role of EVs in regenerative medicine is still partially unknown. For example, can MSCs be regulated by self-secreted EVs? This is crucial given that the therapeutic role of MSCs is based on the ability to rapidly respond to an impaired microenvironment, which is difficult to achieve with exogenous EVs [14]. Besides, the current research on periodontal regeneration induced by EVs is limited to periodontitis, and there is no article reported on the application of non-periodontitis oral disease, such as tooth replantation. Since ADSCs showed positive effects in rat tooth replantation [73], we speculated that EVs, as paracrine products of MSCs, might also have promoting effect on tooth replantation. Whether EVs have different mechanisms for promoting periodontal regeneration in acute injury and inflammatory environments remain to be studied. As another point, most research now focuses on characterizing some selected cargoes and their biological functions. A series of key molecules, especially miRNAs and proteins, are considered to be the active mediators of the regenerative capacity of EVs. Next, the scholars confirm the positive role of these key molecules in tissue regeneration by altering their levels in EVs. Nevertheless, whether one or a few cargos of EVs are sufficient for the therapeutic effects is yet unknown, and wide applications to characterize EVs content and analyze functional activity on a large scale are still lacking [74].

## Application forms of EVs in preclinical studies

**Fig. 2:**
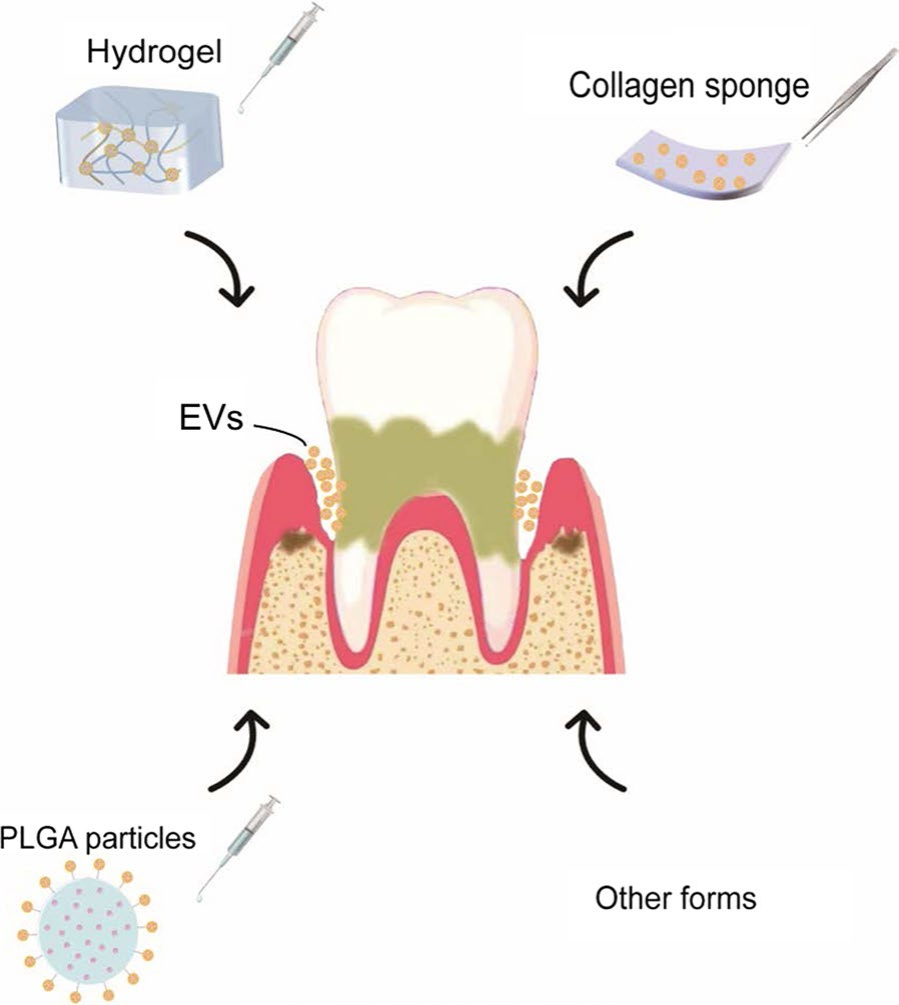
Schematics of commonly used application forms of EVs in periodontal regeneration

As for the application of EVs in vivo, some scholars directly injected MSC-EVs into the defect area of animal models [49, 56, 72], which were proved to have the effect of promoting periodontal regeneration compared with the control group. However, naked EVs are vulnerable to damage in vivo and have a short half-life. EVs' post-application stability and retention are the key obstacles to clinical application. If EVs are directly applied to periodontal defects, oral salivary flow and periodontal pockets' environment of exposure will lead to the rapid release of EVs, making it challenging to play a sustained therapeutic effect. Therefore, the treatment of periodontitis with EVs usually requires high-frequency dosing. EVs administrated into the periodontal pocket using a biocompatible and biodegradable delivery system were found to overcome low tissue residence time and provide a controlled release system to maintain bioactivity [75]. So far, the main approach to address this problem is the delivery system that wraps EVs using different types of hydrogels, collagen sponges, synthetic polymers, and other biomaterials (Fig. 2). However, EVs need to be loaded with these carriers before being used immediately; otherwise, they may be released from the system during storage. Furthermore, the optimal release period for therapeutic EVs to induce tissue regeneration is still unclear. The current EV application forms used for periodontal regeneration are summarized below (Table 2).

**Table 2:** Different EVs application forms used in animals with experimental periodontal defect

Application Forms	Cell type	Animal model	System composition	Key features	References
Hydrogel delivery system	DPSCs	Mouse	CS hydrogel and DPSC-exosome solution	The loading efficiency of exosomes in DPSC-exosome/CS was about 80%, and the system alleviated periodontitis in mice	Shen et al. [58]
	BMSC	Rat	5% gelatin, 10% Laponite and BMSC-sEV	The nanocomposite hydrogel has a sustained release effect on BMSC-sEV without adverse reactions	Liu et al. [59]
	DFSCs	Rat	2.5% laponite, 1.25% gelatin, and 500 μg/ml sEV	The hydrogel system can be released continuously for 21 days without affecting biological activity	Shi et al. [47]
Collagen sponge scaffold loading system	DFSCs	Rat	SC collagen sponge and DFSC-MVs	CS showed a typical spongy structure and regular pore distribution and facilitated adhesion of MVs	Yi et al. [66]
	MSCs	Rat	Bovine I/III collagen sponge and 40 g exosomes	Exosomes combined with collagen sponge promoted periodontal regeneration without any adverse reactions	Chew et al. [70]
	DFSCs	Rat	Collagen sponge and DFSC-sEV	The collagen sponge group formed scattered new bone fragments and are denser than the control group	Ma et al. [71]
Injectable HA/sEV system	DFSCs	Beagle dog	HA system (Gengigel® containing 5% HA) and 200 μg sEV	HA released sEVs significantly promoted the proliferation and migration of PDLSCs, which was the same as that of sEVs alone	Huang et al. [46]
Dual delivery PLGA microparticles	GMSCs	Rat	PLGA microparticles, minocycline and GMSC-EVs	PLGA microparticles system exerts dual effects of antibacterial and periodontal regeneration	Zarubova et al. [53]

### Hydrogel delivery system

Hydrogels are nanometer biomaterials with good biocompatibility and tunability and can achieve minimally invasive drug delivery, so they are commonly utilized in tissue engineering [76]. In regenerative medicine, they are used as scaffolds, barriers, drug delivery platforms, or cell encapsulation matrices. A large number of biopolymer hydrogel-loaded EVs with different chemical structures have been used for in vivo studies. For example, SHEN et al. obtained a 2% CS hydrogel by adding 50% β-glycerophosphate to the 2% chitosan (CS) solution, which was combined with the DPSC-exosome solution soon afterward [58]. They injected the system locally into the periodontal defect of mice. The application of the DPSC-exosomes Composite System sped up the repair process of alveolar bone and periodontal epithelium in periodontitis mice.

According to research, a solid-phase hydrogel network can be formed by 5% gelatin and 2% laponite. So, in the study of Liu et al. [59] and Shi et al. [47], the semi-solid injectable biological scaffold was created using a hydrogel system with Laponite and gelatin. BMSC-sEV hydrogels were injected into the periodontal pockets of rats once a week for 4 or 8 weeks. The results showed that nanocomposite hydrogels are an effective vector and have a sustained release effect on BMSC-sEV without adverse reactions while optimizing their therapeutic performance.

### Collagen sponge scaffold loading system

Collagen sponges are natural extracellular matrix-derived biomaterials that, along with other biomaterials for periodontal regeneration, are widely used for space maintenance [70]. Compared with bone substitutes, collagen sponge has the advantages of good biocompatibility, absorbability, and relative inertness and is an ideal biological scaffold for MSC-EVs [70]. Furthermore, rapid resorption of residual collagen sponges has been reported to be desirable for avoiding the risk of infection and increasing tissue regeneration in periodontal defects [77]. YI et al. cut CSs into 3(long) × 2(wide) × 1(deep) mm blocks and dripped 10^9^ matrix vesicles (MVs), a subtype of EVs, onto them [66]. CS showed a characteristic spongy structure with an orderly framework and regular pore distribution under SEM, and MVs around 100 nm in diameter were observed to adhere to the material. In vivo experiments in rats indicated that MVs loaded with CS collagen sponge had a significant rescue effect on periodontitis and were promoters for alveolar bone regeneration.

In the study of CHEW et al., the bovine I/III collagen sponge was cut into 3(long) × 2(wide) × 1(deep) mm pieces, loaded with 40 μg exosomes, and implanted in rat periodontal defects subsequently [70]. Although exosomes from collagen sponges were rapidly released and decayed during the first 48 h, the effect on periodontal regeneration lasted for at least four weeks. In another study, MA et al. [71] loaded DFSCs-sEVs onto a collagen sponge and transplanted them into the periodontal defect area of rats. The surface of the collagen sponge altered from porous to rough and gritty revealed by SEM. Both studies showed that MSC-EVs combined with collagen sponge potentially stimulated soft tissue and bone tissue regeneration in rat periodontal defect models without creating any negative impacts.

### Other application forms

The ideal mode of application requires the controlled release of MSC-EVs from biomaterials, which can facilitate the recruitment of endogenous stem cells or immune cells and help build a favorable microenvironment for tissue regeneration. Meanwhile, they can enhance tissue revascularization and be degraded and replaced by newly formed tissue. Besides hydrogels, other gels loaded with EVs have also been reported to be applied. As described by Huang et al., they dissolved DFSC-sEVs in PBS and then blended them with Gengigel® (RICERFARMA SRL, Italy), which contained 5% hyaluronic acid gel (HA) [46]. Later, they injected 50 μl of HA system into the periodontal pocket of beagle dogs. After eight weeks, it was found that bone defects were repaired to some extent and there were significantly more Sharpey's fibers perpendicular to the cement-like tissue layer, and the PDL's width and blood vessel density were both enhanced.

Furthermore, polylactic-co-glycolic acid (PLGA) is a biocompatible and biodegradable biomaterial that is widely utilized as a drug delivery system for clinical diagnosis or therapy. Dual delivery microparticles based on PLGA are engineered by ZARUBOVA et al. [53]. PLGA particles made up of half lactide and half glycolide were designed to carry both antibiotics and GMSC-sEVs and to provide sustained release of these two cargos. Microparticles loaded with minocycline as well as GMSC-EVs were injected into the rats' periodontal defect area, and the therapeutic efficacy was assessed after eight weeks. It showed that new alveolar bone formation was evident; the reduction in the distance between the alveolar bone ridge and the enamel junction (CEJ) was significant.

## Clinical applications

Although preclinical studies have evaluated the periodontal regeneration-promoting effect of MSC-EVs on different animal models, clinical trials for periodontitis treatment are currently limited. An Early Phase 1 clinical trial (NCT04270006, Egypt) conducted by Beni-Suef University aimed to investigate the impact of adding ADSCs-derived exosomes to scaling and root planing as a complementary therapy for treating periodontitis. They recruited individuals aged 18 to 50 with advanced periodontitis (stage III or IV) and patients without periodontitis as the control group. However, the specific details and progress have not been updated. No other clinical trials have been documented, while some non-periodontal clinical trials using MSC-EVs for targeted regeneration are reported [74]. More than a dozen clinical trials have been registered on “www.clinicaltrials.gov*”* on the safety and therapeutic application of MSC-EVs for other diseases (Table 3). Among these, only one trial (NCT04493242, US) which applied intravenous administration of BMSCs derived EVs for the treatment of COVID-19 associated acute respiratory distress syndrome (ARDS) has been completed yet. They demonstrated that BMSC-EVs were safe and capable of restoring oxygenation, downregulating cytokine storm, and rebuilding immunity, making them a promising candidate for the treatment of severe COVID-19 [78].

**Table 3:** Clinical trials of MSC-EVs in periodontitis and other diseases

Disease	MSC type	Country	NCT number	Status
**Oral diseases**				
Periodontitis	ADSCs	Egypt	NCT04270006	Unknown
**Trauma**				
Burn wounds	BMSCs	Not shown	NCT05078385	Not yet recruiting
Wound healing	ADSCs	China	NCT05475418	Not yet recruiting
**Gastrointestinal diseases**				
Medically refractory Crohn's disease	Adult allogeneic BMSCs	Not shown	NCT05130983	Not yet recruiting
Medically refractory ulcerative colitis	Adult allogeneic BMSCs	Not shown	NCT05176366	Not yet recruiting
Resistant perianal fistula	Placental mesenchymal stem cell	Iran	NCT05499156	Not yet recruiting
Complex anal fistula	Placental mesenchymal stem Cell	Iran	NCT05402748	Recruiting
**Neonatal-related diseases**				
Preterm neonates at high risk of bonchopulmonary dysplasia	BMSCs	US	NCT03857841	Terminated
Extremely low birth weight infants	MSCs	Russian	NCT05490173	Not yet recruiting
**Organ transplant**				
Abdominal solid organ transplant	BMSCs	Not shown	NCT05215288	Not yet recruiting
**Autoimmune disease**				
Dystrophic epidermolysis bullosa wounds	BMSCs	Not shown	NCT04173650	Not yet recruiting
**COVID-19**				
Mild-to-moderate COVID-19	BMSCs	US	NCT05125562	Not yet recruiting
COVID-19 Moderate-to-Severe ARDS	BMSCs	US	NCT05354141	Recruiting
Post-acute COVID-19 and chronic post-COVID-19 syndrome	BMSCs	Not shown	NCT05116761	Not yet recruiting
COVID-19-associated ARDS	BMSCs	US	NCT04493242	Completed

In addition, some patents on EVs application in the treatment of periodontitis also provide evidence support for clinical translation. Tian et al. (CN202010607634.9) invented and disclosed an injectable pharmaceutical composition incorporating DFSC-derived exosomes, demonstrating that it is effective in treating periodontitis. Another patent by Tian et al. (CN202110910962.0) found for the first time that MSC-derived ApoBDs can promote the formation of new bone and promote bone regeneration, which has a good effect on the treatment of bone defects. Xu et al. (CN202210351949.0) invented and disclosed a mineralized collagen gel loaded with exosomes of GMSCs and a preparation method thereof. The synthetic gel can be injected into the bone defect area under the state of periodontitis and promote local bone repair or bone expansion. Jiang et al. (CN202110125452.2) verified the ability of exosomes from SHEDs to promote the proliferation and osteogenic differentiation of BMSCs and also confirmed that they can enhance alveolar bone repair and regeneration in a periodontitis model. John et al. (US15884921) provided a composition containing stem cell exosomes, which can facilitate PDL repair by inducing type I collagen synthesis in periodontal ligament fibroblasts.

It is well known that EVs have advantages over MSCs in clinical applications, such as stability, immune rejection, ethical supervision, and others. MSC-EVs appear to be the future direction of periodontal regeneration therapy, whereas there are still some inadequacies to be aware of. Firstly, no standardized extracting methods and characterization analysis of EVs have been accepted by all. According to MISEV2018 [30], there is no single optimal extracting method, and the selection of methods should consider the source and application of EVs. In this case, the establishment of standardization is quite difficult. And the low yield of EVs makes it challenging to achieve EVs' industrialization and mass production. The relevant mechanisms currently understood still need to be integrated and deepened. Furthermore, problems concerning the clinical application, including safety, application forms, dosage and frequency, pharmacokinetics, side effects, etc., remain to be investigated. Before clinical application, good quality control is essential to ensure the safety and therapeutic efficacy, in which identification of the quantity, size, identity, and purity of EVs should be included [79].

The selection of EV sources is also worth discussing in clinical applications. EVs derived from different MSCs mostly share common features, such as promoting angiogenesis, enhancing tissue repair, and inhibiting inflammation, but their mechanisms are slightly different [80]. In general, MSC-EVs have certain similarities with parental MSCs because the content they carry is from their parental cells. Sometimes in treating a specific disease certain disease, one type of MSC-EVs shows better therapeutic effects than others [81, 82]. However, EVs derived from which source of MSC is more effective for periodontal regeneration are still unknown.

## Conclusion and prospects

As discussed above, the good regeneration-promoting effects of MSC-EVs in the treatment of periodontitis have been confirmed in both in vivo and in vitro experiments. It is achieved from the aspects of inhibiting oxidative stress, regulating immune response, and promoting tissue repair. Moreover, varied application methods of EV delivery are applicated without affecting their therapeutic effects.

In a word, MSC-EVs therapy is a promising method for periodontal regeneration. Still, more mechanism studies and clinical trials are yet to be carried out before they can be used in the clinical treatment of patients with periodontitis.

## Data Availability

Not applicable.

## Abbreviations

MSCs: Mesenchymal stem cells
EVs: Extracellular vesicles
MSC-EVs: MSCs-derived extracellular vesicles
PDL: Periodontal ligament
GTR: Guided tissue regeneration
PDLSCs: Periodontal ligament stem cells
DFSCs: Dental follicle stem cells
DPSCs: Dental pulp stem cells
BMSCs: Bone marrow stem cells
ADSCs: Adipose tissue-derived stem cells
DMSO: Dimethyl sulfoxide
MVs: Microvesicles
ApoBDs: Apoptotic bodies
MVBs: Multivesicular bodies
ESCRTs: Endosomal sorting complexes required for transport
ILVs: Intraluminal vesicles
ROS: Reactive oxygen species
GSK-3β: Glycogen synthase kinase-3β
RANKL: The receptor activator of NF-κB ligand
OPG: Osteoprotegerin
GMSC: Gingival mesenchymal stem cells
Mø: Macrophage
M1: The pro-inflammatory phenotype
M2: The anti-inflammatory phenotype
Th17: T-helper 17
Treg: Regulatory T
SHED: Stem cells from exfoliated deciduous teeth
HUVEC: Human umbilical vein endothelial cells
VEGF: Vascular endothelial growth factor
VEGFA: Vascular endothelial growth factor A
CRMVs: Collagenase-released MVs
CS: Chitosan
MVs: Matrix vesicles
HA: Hyaluronic acid gel
PLGA: Poly lactic-co-glycolic acid
CEJ: The alveolar bone ridge and the enamel junction
CAL: Clinical attachment level
ARDS: Acute respiratory distress syndrome
